# Referral patterns and patient characteristics observed during the first year of Ireland’s inaugural community hub diabetes service: a retrospective analysis

**DOI:** 10.1007/s11845-025-03975-8

**Published:** 2025-07-19

**Authors:** Katie De Jong, Tommy Kyaw-Tun, John H. McDermott, Seamus Sreenan, Colin Davenport

**Affiliations:** 1 Department of Academic Endocrinology, Connolly Hospital Blanchardstown, Dublin, Ireland; 2 The Royal College of Surgeons in Ireland Medical School, Dublin, Ireland

**Keywords:** Chronic disease management, Community-based care, Hospital waiting lists, Ireland, Referral patterns, Type 2 diabetes

## Abstract

**Background:**

Enhanced Community Care (ECC) for type 2 diabetes mellitus (T2DM) in Ireland introduces episodic, community-based, consultant-led multidisciplinary care of diabetes that in turn supports the general practitioner (GP)-delivered chronic disease management programme (CDMP). The Dublin North West (DNW) hub, Ireland’s first fully operational ECC hub, began providing its diabetes service in March 2023.

**Aims:**

This study reviewed the first year of the DNW hub’s diabetes operations, focusing on referral patterns, patient demographics, clinical characteristics, and CDMP eligibility (only those people living with T2DM with a medical card or a GP visit card are eligible for CDMP for T2DM; otherwise, people living with T2DM must self-pay for GP-provided T2DM care).

**Methods:**

A retrospective analysis was conducted on patient charts and hospital databases for all referrals to the DNW hub from March 2023 to March 2024.

**Results:**

Out of 204 referrals, 67% were redirected from hospital waiting lists, 22% were direct GP referrals, and 11% were internal from other hub services. The average wait time from referral to first appointment was 8.6 weeks. Attendees were 44% female and 56% male, with an average age of 56.2 years. Notably, only 46% were eligible for CDMP. During the study period, the number of people living with T2DM waiting for diabetes appointments at Connolly Hospital decreased by 61%, with the average waiting time reduced from 11 to 5 months.

**Conclusions:**

The first year of activity in the DNW hub illustrates the potential of the ECC model to positively impact hospital waiting lists. Our data also suggests that eligibility to access the CDMP may merit expansion as part of the ongoing implementation of ECC in Ireland.

## Background

Diabetes is one of the greatest public health challenges of the twenty-first century [1]. In Ireland, as elsewhere, the prevalence of type 2 diabetes mellitus (T2DM) is increasing. As reported by Feeney et al., 10% of adults over the age of 50 and 16% of those over 80 in Ireland are affected by T2DM [2]. Projections suggest a 60% rise in the number of adults with T2DM in Ireland over the next 10–15 years [3]. This surge in T2DM poses a significant burden on the Irish healthcare system [4]. In 2019, the financial toll of diabetes care in Ireland was estimated to be between €1.2 and €1.4 billion, with approximately 50% of these costs associated with complications and hospitalizations [4].

In response to this escalating health crisis, Ireland is currently undergoing a transformative phase in diabetes care [5]. The Enhanced Community Care (ECC) programme, as part of the Sláintecare initiative, aims to enhance community healthcare services across the country, in particular by establishing consultant-led, multidisciplinary specialist teams that are based within integrated care centres (or hubs) and who deliver care of chronic diseases [5]. These specialist teams support the management of T2DM outside of the hospital system, assisting general practitioners (GPs) and nurse practitioners in managing complex diabetes and its associated comorbidities when required [6]. In contrast to the traditional approach in the hospital outpatient departments, the T2DM care delivered by the specialist team in the hub is episodic in nature, with people living with T2DM typically discharged to the chronic disease management programme (CDMP) with their GP once stabilised and/or optimised from a T2DM perspective. As part of the wider effects of the Sláintecare programme, it has been envisioned that this approach will reduce the need for hospital outpatient clinic attendance in Ireland, thus alleviating pressure on the hospital system and reducing the long-term healthcare costs associated with T2DM [6].

The first integrated care centre in Ireland to fully implement this novel paradigm of T2DM care was the integrated care centre in Community Healthcare Organization Dublin North City and County, Dublin North West (CHO DNCC DNW), linked to Connolly Hospital, Blanchardstown. Operational since March 2023 and with a catchment area of approximately 150,000 people in DNW, this hub is located in Cuan Aoibheann in St. Mary’s hospital in the Phoenix Park and accepts referrals from the three community health networks (CHNs) of Blanchardstown, Blakestown and Finglas. The hub’s specialist team includes a consultant endocrinologist (CD), a candidate advanced nurse practitioner in diabetes, diabetes nurse specialists, podiatrists, dieticians and administration support. The hub’s team redirects clinically appropriate hospital referrals for T2DM care to the hub, transfers people living with uncomplicated T2DM from hospital review clinics to the hub and accepts referrals from GPs and other specialties within DNW directly to the hub. In accordance with the chronic disease model of care (MOC), integration of patient services between the community and the hospital was supported by a number of factors, including the consultant endocrinologist post being split between the acute hospital and the hub, the presence of GP representatives within the hub management structure, and a local governance committee which has both acute hospital and community representation [6].

As this is a novel model of care, heretofore, there have been no studies examining the activities and impact of the hubs and their specialist teams on T2DM care in Ireland. Accordingly, the present study was conducted with the aims of analysing referral patterns to the DNW hub during its first year of activity, along with an exploration of hub patient demographics and clinical characteristics, CDMP eligibility rates and the impact of hub activity on acute hospital waiting times.

Understanding the impact of the ECC on diabetes management in Ireland is crucial, not only for local healthcare policy, but also for its implications in the global context of diabetes care. As diabetes prevalence continues to rise worldwide, innovative models of care, such as those implemented in the community setting in Ireland, offer insights for other countries dealing with similar challenges. As the first study to report on clinical activity and patient characteristics attending a hub for T2DM in Ireland, this research was conducted in order to contribute to regional and national service planning and also to add to the broader discourse on integrated care and its potential benefits for healthcare systems via community-centric approaches.

## Methods

### Study design and setting

This retrospective study analysed data relating to T2DM care from the DNW hub, an integrated care centre that provides specialist chronic disease care to people living with T2DM who live in the Blakestown, Blanchardstown and Finglas community health networks, and which is linked to Connolly Hospital Blanchardstown.

### Study population

The study cohort comprised of people living with T2DM referred to the consultant-led community diabetes hub in DNW during the initial 12 months (March 2023–March 2024) of its operation.

### Data collection

Data were collected from a number of different sources.

#### Referral data

Referral letters from 204 people living with T2DM were reviewed, and data was extracted. This included people living with T2DM referred directly to the hub by GPs, re-directed referrals from the hospital waiting lists, referrals from the diabetes clinics in the hospital and internal referrals within the hub. All data extracted were from people living with T2DM who were offered an outpatient appointment in the Cuan Aoibheann community hub between March 2023 and March 2024. Data collected from referral letters included the source of referral, date of referral and urgency of referral. Referral letters redirected from the general diabetes waiting list in Connolly hospital to the community hub were screened for the date of redirection from the general waiting list to the community hub. Wait times for the first appointment in the hub were calculated based on either the date of the initial direct referral or from the date of redirection from the general waiting list in Connolly hospital.

### Administrative data

Data extracted from the community hub’s administration system included the number of people living with T2DM who did not attend or cancelled their first hub appointment. People living with T2DM who did not attend their first appointment were offered a second appointment at a later date. The wait time was calculated from the date that the first appointment was offered to the date that the first appointment was attended.

### Clinical data

A chart review was conducted in order to extract clinical and demographic data from the patient’s first clinic appointment (for those who attended). A standardised dataset was recorded including age, gender, current eligibility for the CDMP, HbA1c, lipid profile, urine albumin:creatinine ratio, blood pressure, body weight, body mass index, whether the patient required a referral to the retinal screening programme (indicated for all people living with T2DM with diabetes), whether the patient was enrolled in the long-term illness (LTI) scheme and diabetic foot risk status. The latter was assessed by the community podiatrist in keeping with the diabetes foot model of care [7]. People living with T2DM were noted as eligible for the CDMP if they had a medical card or a GP visit card (a specific question for all new attendees in the hub) or if they were over 70 (all people living with T2DM over 70 can obtain a GP visit card). The diabetes administration team in Connolly hospital tracked wait times for outpatient appointments in Connolly hospital, and these data were extracted from the diabetes administration data base.

### Statistical analysis

The number of direct referrals to the hub and redirected referrals were calculated using frequency analysis. Descriptive statistics were conducted to assess wait times. Patient attendance and cancellation rates for people living with T2DM who did not attend their first appointment were calculated as a percentage. Categorical variables such as gender, eligibility for chronic disease management and podiatry risk status were summarised using frequency tables. Descriptive statistics including mean and standard deviation were used for analysis of lab results and clinical measurements including HbA1c, lipid profile, urine albumin:creatinine ratio, blood pressure, body mass index and weight. Visual representations of the above were conducted using box graphs.

## Results

### Patient demographics

Of 204 participants in this study, 115 (56%) were male and 89 (44%) were female. The average age of participants was 56.2 with a standard deviation of 12.1 years.

### Referral patterns

Among the 204 referrals received by the consultant-led diabetes service in the hub, 168 were designated as “routine” with 35 marked as “urgent” and one unknown. With regard to the source of the referrals, 137 (67.2%) of the 204 people living with T2DM offered a first appointment in the community hub which had been redirected from the waiting list of the T2DM clinic in Connolly hospital (of which 125 had come from GPs, 6 from other services in Connolly hospital, 1 referral from a private hospital in Dublin, 1 from the National Orthopaedic Hospital in Dublin and 4 were from nurse practitioners in the community). The remaining 67 (32.8%) referrals had been sent directly to the consultant-led diabetes service in the hub, with 45 of these coming from GPs, 18 coming from other services within the hub (14 from diabetes clinical nurse specialists, 2 from the podiatry service, 2 referrals from dieticians), 2 came from practice nurses in the community and 2 came from acute hospital consultants within Connolly hospital.

### Wait times and attendance

The overall wait time for the first hub appointment, including both direct and redirected referrals, was 8.6 weeks with a standard deviation of 4.6 weeks (Table 1). Among people living with T2DM referred directly to the hub, the average wait time was 7.1 weeks with a standard deviation of 4.1 weeks (Table 1). Among people living with T2DM redirected from the Connolly hospital diabetes waiting list, the average wait time was 9.3 weeks with a standard deviation of 4.7 (Table 1). A total of 19.6% of people living with T2DM did not attend their first appointment without informing the hub beforehand and 3% cancelled their first appointment. Of the people living with T2DM who did not attend their first appointment, 70% had been redirected from Connolly hospital. In total, 167 attended their initial appointment.

**Table 1:** Wait times of people living with T2DM attending the hub

**Wait times**	Mean (standard deviation)
Overall wait time	8.6 weeks (4.6)
Redirected from acute hospital waiting lists	9.3 weeks (4.7)
Direct referral to the hub	7.1 weeks (4.1)

The total numbers of people living with T2DM waiting for a new diabetes appointment in Connolly Hospital decreased by 61% over the time period studied (from 262 to 101), with the average waiting time for these appointments decreasing from 11 to 5 months.

### Clinical characteristics

With regard to the clinical and demographic characteristics of the 167 people living with T2DM who attended a first appointment in the hub, 94 were male and 73 were female. The mean age was 55.2 years with a standard deviation of 11.79 years. The main clinical characteristics of these people are documented in Table 2. Table 3 details the diabetes medications they were taking at the time of their first attendance at the hub, with the majority (*n* = 133, 79.6%) of those referred to the hub taking metformin, and SGLT-2 medications representing the second most commonly utilised treatment for diabetes (*n* = 50, 29.9%).

**Table 2:** Laboratory values, blood pressure, BMI and podiatry risk status at time of first visit to hub

**Lab values**	Mean (standard deviation)
HbA1c	64 mmol/mol (20.4)
Total cholesterol	5.15 mmol/l (4.7)
LDL	2.58 mmol/l (1.05)
HDL	1.76 mmol/l (4.86)
Triglycerides	2.20 mmol/l (1.23)
eGFR	77.13 mL/min/1.73m^2^ (16.50)
uACR	18.19 mg/mmol (41.10) (Median = 2.4)
**Blood pressure and BMI**	
Systolic BP	139.2 mmHg (14.8)
Diastolic BP	83.5 mmHg (11.1)
BMI	32.4 kg/m^2^ (8.00)
Weight	93.1 kg (23.2)
**Podiatry risk status**	
High	18.56%
Moderate	27.54%
Low	52.69%
Not assessed	1.21%

**Table 3:** Baseline diabetes medications at time of first hub attendance

Medication class	Number of people living with T2DM prescribed class of medication at time of first hub attendance
Metformin	133 (79.6%)
SGLT2	50 (29.9%)
DPP-IV	25 (14.9%)
GLP-1	20 (11.9%)
Gliclazide	19 (11.3%)
Insulin	13 (7.7%)

### Other aspects of T2DM care

Of the 167 people living with T2DM who attended the hub for a first appointment, 74 (46%) were noted to be eligible for GP-delivered CDMP for their T2DM, with the remainder needing to self-pay for GP visits. For 7 people living with T2DM, there was no documentation regarding their CDMP eligibility. Of note, 55 people living with T2DM (33%) required a referral to the retinal screening programme and 35 (20%) had not submitted an application for the LTI scheme at the time of their first attendance in the hub.

## Discussion

The findings from this study of the inaugural year of the first fully functional hub delivering ECC for T2DM in Ireland offer a number of novel insights into the potential benefits and challenges that may be associated with this model of care as it is expanded across the country.

With regard to the impact of the hub’s activities on the acute hospital, we observed a significant 61% reduction in the number of people living with T2DM waiting for T2DM appointments at Connolly Hospital during the initial year of hub activity, with the average waiting time decreasing from 11 to 5 months. We submit that this substantial reduction demonstrates the potential of community hubs to facilitate access to specialist diabetes care and reduce hospital wait times. Similar outcomes have been observed in other countries where community-based care models have been implemented, reinforcing the efficacy of this approach [8, 9], but our data are entirely novel for Ireland.

When referral patterns were analysed, our study population were noted to have a higher prevalence of male people living with T2DM and an average age of 56.2 years, consistent with national data on T2DM [4]. The majority of referrals (67%) to the DNW hub were redirected from the hospital waiting lists, once again consistent with a successful initial implementation of the hub’s objective to alleviate pressure on hospital services. The remaining referrals came primarily from GPs (22%), which may suggest a willingness for to utilise and engage with the hub by primary care providers.

An additional significant finding of this study is the relatively low rate of eligibility for the CDMP among people living with T2DM referred to the hub. It should be noted that people living with T2DM who do not meet eligibility for the CDMP need to self-pay if they attend their GP for T2DM. In general, people living with T2DM with T2DM are eligible for the CDMP if they hold a medical card or a GP visit card (available to all over 70). Only 46% of the people living with T2DM referred to the hub in this study were eligible for the CDMP, highlighting a significant gap in eligibility to state-funded treatment in the community and a potential challenge for ECC.

This study identified a non-attendance rate of 19.6% for a first appointment in the community hub for T2DM. This is higher than the national average of 13% and represents a significant direct cost to the healthcare service and a missed opportunity to provide specialist patient care [10]. We do note that people living with T2DM who were redirected from the hospital waiting list to the community were sent written communication indicating that this action was taking place (with the goal of diminishing any confusion around the redirection process). Furthermore, during the study timeframe, all people living with T2DM who did not attend their first appointment in the hub received an offer of a second appointment, and contact information for the hub was provided with all appointment letters. Nonetheless, we submit that significant improvements are desirable in this area. It may be significant, in this context, that the DNW community hub has lesser access to public transport in comparison to Connolly Hospital, and this may have created a barrier for individuals without access to a car. Addressing barriers such as this through local infrastructure development and the offering of telehealth options may help to support patients in attending the hub for their appointments in the future. Finally, we note that specialist diabetes care within community hubs, in general, is new to Ireland, and it is possible that increased awareness among the public of what services are provided within the hubs will also lead to improved engagement and attendance rates over time.

The clinical data in this study revealed that a significant portion of people living with T2DM required further interventions and/or support at their time of first review by the specialist services in the community hub. This included a significant need for referral to the retinal screening services (33%) and submitting an application for the LTI scheme (20%). According to the ICGP Guide to Integrated Type 2 Diabetes, all people living with T2DM diagnosed with type 2 diabetes should be registered with the National Retinal Screening Programme and the LTI scheme [11]. This underscores the hub’s crucial role in identifying and addressing unmet clinical needs in people with diabetes, and these findings also suggest a possible need for the community hub to educate and support primary-care delivered diabetes care such that these registrations take place without the need for specialist-led care in a greater percentage of cases.

The clinical data also indicated that a substantial portion of people living with T2DM had suboptimal glycaemic control (65.6% had a HbA1c of 53 mmol/mol or greater) on first presentation to the hub, with a mean HbA1c level of 64 mmol/mol (8%). According to the 2024 ADA guidelines, the target HbA1c for most adults with diabetes is less than 53 mmol/mol (7%). The mean LDL cholesterol level was 2.58 mmol/l, likely indicating suboptimal lipid control at time of referral in this cohort. According to ADA guidelines, people living with T2DM with diabetes and atherosclerotic cardiovascular disease (ASCVD) should aim for LDL cholesterol levels below 1.4 mmol/l. If there is no history of ASCVD, then they should aim for an LDL of < 1.8 [12]. Blood pressure control in this cohort was also noted to have scope for improvement, with the ADA recommending a target of less than 130/80 mmHg for most adults with diabetes, whereas the mean systolic and diastolic blood pressures observed in the cohort were 139.2 mmHg and 83.5 mmHg, respectively [12]. Finally, the mean BMI of 32.37 kg/m^2^ and mean weight of 93.1 kg indicated a high prevalence of the disease of obesity within this cohort. The ADA emphasises the importance of weight management through lifestyle modifications, pharmacotherapy and metabolic surgery to improve glycaemic control and reduce cardiovascular risk [12], and our data suggests that access to these interventions will be required as part of T2DM care within the community.

In general, the failure to initiate or intensify therapy when necessary is often considered a significant barrier to achieving optimal disease control in T2DM, and our data suggests that a significant part of this cohort was in need of treatment intensification at the time of their first attendance in the hub. Healthcare professionals who are not specialists in diabetes care may face competing priorities, time constraints and uncertainty about newer therapies that affect treatment intensification decisions. It is possible that initiatives like the community hub may help address such challenges by providing targeted education, decision support and opportunities for rapid referral. This, however, will need to be examined in future research into this field.

The podiatry risk assessment revealed that 18.56% of people living with T2DM were classified as high risk, 27.54% as moderate risk, and 52.69% as low risk for foot complications. The Diabetic Foot Model of Care Guidelines in Ireland stress the importance of regular foot examinations and appropriate interventions to prevent severe complications such as ulcers and amputations [7]. The hub’s focus on podiatry care underscores the critical need for integrated services to manage diabetes effectively and prevent long-term complications.

This study has significant limitations, including its retrospective design and (with regard to referral patterns) its reliance on referral letter data which may not capture all relevant clinical details. Future research should include prospective studies with larger sample sizes and longer follow-up periods to fully assess the long-term impact of the ECC model on diabetes management and health outcomes and include comparison with primary-care managed people living with T2DM and the standards of care within hospital clinics. Additionally, exploring patient experiences and satisfaction could provide deeper insights into the barriers to attendance and adherence to treatment plans. Finally, we note that as a new model of care in Ireland, this is a rapidly changing area and that repeated cycles of feedback and refinement are ongoing. It is important to note that the present study does not compare the nascent activities of the hub against those of hospital diabetes services, and it is probable that additional resourcing of hospital diabetes services would also improve waiting lists. What the present study does, however, is examine the effects of hub activities as a novel paradigm of T2CM care, within the greater context of Slaintecare and the movement of chronic disease care to the community.

## Conclusion

To conclude, this retrospective analysis of the inaugural year of Ireland’s first community hub diabetes service in DNW reveals several key insights into the possible opportunities and challenges of implementing community-based diabetes care. We identified an immediate and significant benefit for hospital services, with Connolly Hospital experiencing a reduction in average waiting times for T2DM from 11 to 5 months. The study, however, also highlighted several areas that require further attention, in particular a low eligibility rate for the CDMP.

Overall, the successful implementation of the DNW hub highlights the potential of the ECC model to enhance diabetes care delivery in Ireland. We submit that our findings provide initial support for the ongoing implementation of this model across the country.
